# Generating Different Polarized Multiple Vortex Beams at Different Frequencies from Laminated Meta-Surface Lenses

**DOI:** 10.3390/mi13010061

**Published:** 2021-12-30

**Authors:** Pengfei Gao, Rui Yang

**Affiliations:** National Key Laboratory of Antennas and Microwave Technology, School of Electronic Engineering, Xidian University, Xi’an 710071, China; pengfeigao@stu.xidian.edu.cn

**Keywords:** meta-surface lens, orbital angular momentum, multiple beams, polarization

## Abstract

We demonstrate the generation of multiple orbital angular momentum (OAM) vortex beams with different radiating states at different frequencies through a laminated meta-surface lens consisting of a dual polarized meta-array interconnected with a frequency selective meta-array. The co-linearly polarized (LP) waves from the source can directly penetrate the meta-surface lens to form multiple OAM vortex beams at one frequency. On the other hand, the meta-surface lens will be capable of releasing the cross-LP counterparts at another frequency with high-efficient polarization conversions to have multiple OAM vortex radiations with different radiating directions and vortex modes. Our design, using laminated meta-surface lens to synthesize multiple OAM vortex beams with orthogonal polarizations at different frequencies, should pave the way for building up more advanced vortex beam communication system with expanded diversity of the meta-device.

## 1. Introduction

Orbital angular momentum (OAM) vortex beams, with the special intensity distributions and spiral phase fields, have demonstrated great potential to improve the spectral efficiency in the wireless communication [1,2,3,4,5,6,7] due to the good orthogonality between different topological charges of the radiating mode. In particular, generating multiple OAM vortex beams should further expand the coverage of wireless connections by producing additional available channels.

Meta-surfaces have been proved to be an efficient way to generate OAM vortex beams through properly arranging sub-wavelength meta-atom arrays [8,9,10,11,12,13,14,15,16], where multiple OAM vortex beams with controllable beam directions and topological charges have been synthesized [17,18,19,20,21,22]. On the other hand, meta-surfaces have also demonstrated the great capacity of polarization conversions [23,24,25,26,27,28], where linearly polarized (LP) waves are shown to be transformed into their cross counterparts through re-assigning the amplitudes of co- and cross-polarized components. However, most of these works of literature can solely operate at a single frequency with specific wave modulations as they are composed of single-formed meta-atom array. On the other hand, hybrid meta-unit arrays forming the dual-band and multiband meta-surfaces are shown to be capable of generating different functionalities at different frequencies. Clearly, it would offer a great way of multiplexing to expand the channel capacity if the polarization states of multiple OAM vortex beams could be specifically prescribed and re-produced using the same generator at different frequencies. Based on these considerations, we demonstrate the generation of different polarized multiple vortex beams at different frequencies from laminated meta-surface lenses consisting of dual polarized meta-arrays interconnected with frequency selective meta-arrays. We will demonstrate that such laminated meta-surface lenses can achieve multiple OAM vortex radiations with orthogonal polarized states at dual frequencies when we impose an appropriate phase distribution and the radiating OAM vortex beams in each band can be controlled independently since the non-interfering phase modulation of the dual meta-atom arrays.

## 2. Design and Numerical Results

**Figure 1:**
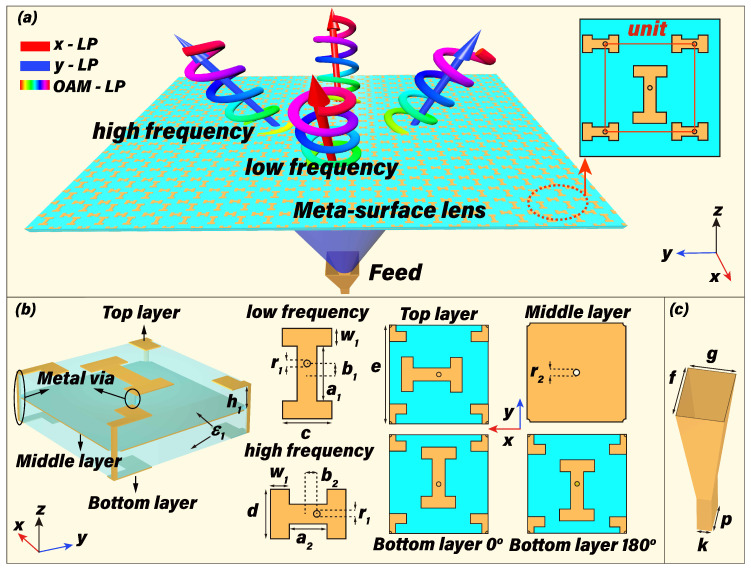
Illustration of the different polarized OAM vortex beams at different frequencies through the laminated meta-surface lens. (**a**) the schematic showing of the dual-band meta-surface lens for different polarized OAM vortex beams. Structural information of the periodic meta-atoms (**b**) and the feed (**c**). The parameters $b_{1}$, $b_{2}$, *c* and *d* are the variables for achieving phase modulations, where $b_{1}$ and $b_{2}$ refer to the distances between the patch center and the metal vias.

Figure 1 schematically demonstrates the generation of different polarized OAM vortex beams at different frequencies through the laminated meta-surface lens with an aperture size of $192\times 200\;{\mathrm{mm}}^{2}$ and the structural parameters are listed in Table 1. The laminated meta-surface lens is composed of the top dual-LP transmitting meta-array, metallic middle-layer partition with etched circular holes, and bottom LP receiving meta-array, where the meta-cells over the top and bottom layer are connected by metallic vias through holes of the metal partition. The periodic LP meta-cells etched over the bottom layer consist of two types of I-shaped patches with different physical dimensions arranged along the *y*-direction, which makes the meta-surface lens only be able to receive the *y*-polarized waves with dual-band frequency response. The gradient dual-LP meta-arrays over the top layer would be selectively excited to realize different radiations at dual frequencies of 14 GHz and 18 GHz, when the *y*-polarized waves at the specific frequency received by the meta-array over the bottom layer are coupled to the corresponding meta-arrays over the top layer through the metallic vias. A rectangular horn functioning as a source is placed $F_{1}=130\;\mathrm{mm}$ away from the lens whose operating bandwidth is from 12 to 18 GHz, and gains are 12.2 dBi at 14 GHz and 14.3 dBi at 18 GHz with 3 dB beamwidth of $46^{\circ }$ and $36^{\circ }$, respectively. Multiple OAM vortex beams with different radiating states at dual frequencies will thus be generated from such laminated meta-surface lens by providing the proper phase gradients. According to the generalized Snell’s laws, the dual-band phase distributions provided by the meta-surface lens can be calculated as

(1) $$\Phi \;=\;k\cdot s+\phi +\phi_{0}$$

where *k* refers to the wave number in the free space, *s* refers to the path of the light to be compensated, ϕ refers to the additional phase to produce OAM vortex beams, and $\phi_{0}$ refers to an arbitrary phase constant that can adjust the phase distributions freely. The phase of the propagation path to be compensated can be expressed as $k^{\prime }\sqrt{x^{2}+y^{2}+{F_{1}}^{2}}$ with $k^{\prime }$ and (x,y) referring to the wave number and the coordinate points of patch at 14 GHz or 18 GHz. The meta-surface lens should introduce the phase gradient as $\arg(\sum_{i=1}^{m}e^{j[k^{\prime }\left(x\sin\theta_{i}\cos\varphi_{i}+y\sin\theta_{i}\sin\varphi_{i}\right)]})$ to form an isophase front in the radiating direction, where *i* refers to the number of the radiating beams, $\theta_{i}$ and $\varphi_{i}$ refer to the travelling directions of each beam. In addition, we also have to impose the orbital angular momentum to each isophase front with $\arg(\sum_{i=1}^{m}e^{j[l_{i}\arg\left(x+jy\right)]})$, where $l_{i}$ refers to the topological charge of each OAM vortex beam. As a result, the whole phase compensations would thus follow:

(2) $$\Phi_{}\left(x,y\right)=k\sqrt{x^{2}+y^{2}+{F_{1}}^{2}}-\arg\left(\sum_{i=1}^{m}e^{j[k\left(x\sin\theta_{i}\cos\varphi_{i}+y\sin\theta_{i}\sin\varphi_{i}\right)+l_{i}\arg\left(x+jy\right)]}\right)+\phi_{0}$$

We can easily acquire the required compensation phase to create the meta-surface lens once the radiation characteristics of the OAM vortex waves in the two bands are given.

**Figure 2:**
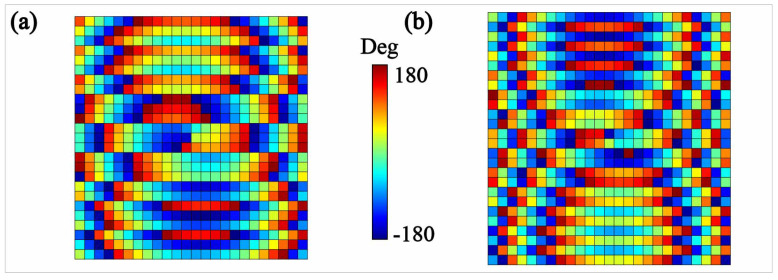
The required phase distributions of the meta-surface lens at 14 GHz (**a**) and 18 GHz (**b**).

Figure 2 demonstrates the corresponding phase distributions of the meta-surface lens calculated by Equation (2). The phase distribution at 14 GHz in Figure 2a includes the superposed phase patterns of the two vortex beams with elevation angle $\theta_{1,2}=\left[30^{\circ }\;30^{\circ }\right]$, azimuth angle $\varphi_{1,2}=\left[90^{\circ }\;270^{\circ }\right]$ and topological charges $l_{1,2}=\left[1\;1\right]$. On the other hand, the phase distribution at 18 GHz in Figure 2b makes the meta-surface lens possess the ability to synthesize the *y*-polarized OAM vortex beams with $\theta_{1,2}=\left[30^{\circ }\;30^{\circ }\right]$, $\varphi_{1,2}=\left[90^{\circ }\;270^{\circ }\right]$ and $l_{1,2}=\left[1\;1\right]$.

**Figure 3:**
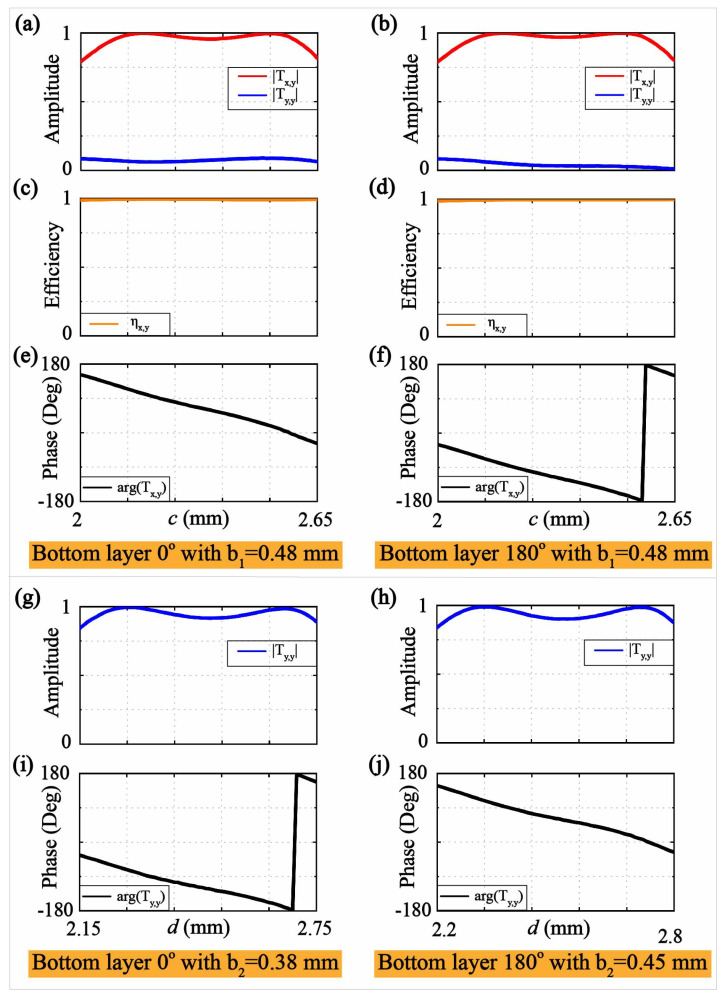
The electromagnetic responses from the meta-unit. The relationships between the dimension *c* of the periodic meta-units and transmitting amplitudes $\left|T_{x,y}\right|$ (**a**,**b**), polarization conversion efficiency $\eta_{x,y}$ (**c**,**d**) and transmitting phases $\arg\;\left(T_{x,y}\right)$ (**e**,**f**) at 14 GHz. The relationships between the dimension *d* of the periodic meta-units and transmitting amplitudes $\left|T_{y,y}\right|$ (**g**,**h**) and transmitting phases $\arg\;\left(T_{y,y}\right)$ (**i**,**j**) at 18 GHz.

Figure 3 demonstrates the transmission performance of the meta-unit with different structural parameters. We can observe in Figure 3a,b that the transmitting amplitudes $\left|T_{x,y}\right|$ of the meta-surface lens atom array would keep a high-level transmission at 14 GHz and the transmitting amplitudes $\left|T_{y,y}\right|$ would always be near 0. The cross-polarization conversion efficiency is defined by ηx,y=Tx,y2Tx,y2Ty,y2+Tx,y2Ty,y2+Tx,y2, where $\left|T_{y,y}\right|$ and $\left|T_{y,y}\right|$ are the transmitting amplitudes of the co-and cross-polarized components. We can observe in Figure 3c,d that the periodic meta-surface lens units possess more than 98% polarization conversion efficiency $\eta_{x,y}$ at 14 GHz. We can observe in Figure 3e that the transmitting phases $\arg\left(T_{x,y}\right)$ change from $-29^{\circ }$ to $152^{\circ }$ when the *c* varies from 2 mm to 2.65 mm, and we can also observe in Figure 3f that the transmitting phases $\arg\left(T_{x,y}\right)$ can cover $\left[-30^{\circ },-180^{\circ }\right]$ and $\left[151^{\circ },180^{\circ }\right]$ when the state of the patch over the bottom layer changes. In the meantime, we can observe in Figure 3g,h that the transmitting amplitudes $\left|T_{y,y}\right|$ of the meta-surface lens unit would maintain the same quality transmission at 18 GHz. We can observe in Figure 3i that the transmitting phases $\arg\left(T_{y,y}\right)$ can cover $\left[-33^{\circ },-180^{\circ }\right]$ and $\left[157^{\circ },180^{\circ }\right]$ when the *d* varies from 2.15 mm to 2.75 mm. The transmitting phases $\arg\left(T_{y,y}\right)$ change from $-26^{\circ }$ to $148^{\circ }$ when *d* varies from 2.2 mm to 2.8 mm, as shown in Figure 3j. As a result, the employed periodic unit cells can release orthogonally polarized electromagnetic fields at 14 GHz and 18 GHz, respectively, while maintaining the high-level transmission at both frequencies. Since the whole meta-surface lens is very thin (with about one-tenth of the wavelength for the operating frequencies) and has a high-level transmission, all the power received by the bottom layer could be radiated by the top layer. In addition, the gradient meta-cells can also possess nearly 360° transmitting phases, thus fulfilling the design purpose for the generation of OAM vortex beams in both bands.

**Figure 4:**
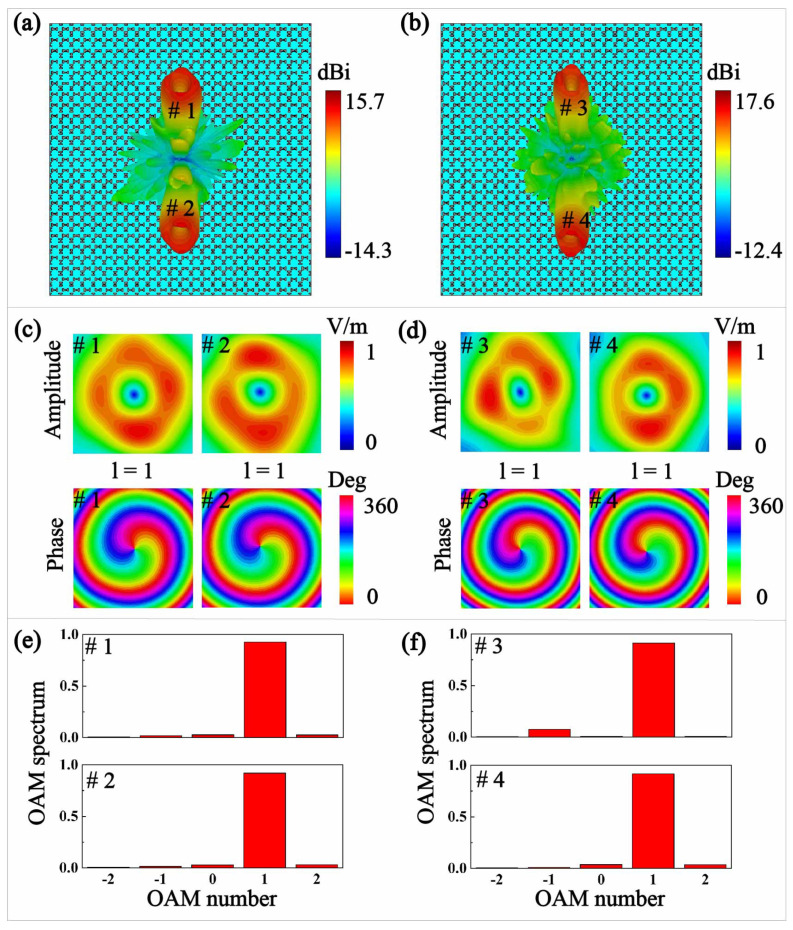
The dual vortex beams with different polarization states from the laminated meta-surface lens at different frequencies. (**a**) the 3D far-field patterns of x-polarized vortex radiations in $\theta_{1,2}=\left[30^{\circ }\;30^{\circ }\right]$ and $\varphi_{1,2}=\left[0^{\circ }\;180^{\circ }\right]$ at 14 GHz; (**b**) the y-polarized 3D far-field patterns of the dual vortex beams in $\theta_{1,2}=\left[30^{\circ }\;30^{\circ }\right]$ and $\varphi_{1,2}=\left[90^{\circ }\;270^{\circ }\right]$ at 18 GHz. The amplitude patterns and phase patterns of *x*-polarized vortex beams at 14 GHz (**c**) and *y*-polarized vortex beams at 18 GHz (**d**). The mode purities of *x*-polarized vortex beams at 14 GHz (**e**) and *y*-polarized vortex beams at 18 GHz (**f**).

The full wave simulations (CST Microwave Studio) are then performed to verify the radiation performances of the laminated meta-surface lens as shown in Figure 4. We can observe in Figure 4a that desired *x*-polarized beams with radiation characteristics of ${\left[\begin{array}{c} \theta_{i}\\ \varphi_{i}\\ l_{i} \end{array}\right]}_{i=1,2}=\left[\begin{array}{cc} \begin{array}{c} 30^{\circ }\\ 90^{\circ }\\ 1 \end{array} & \begin{array}{c} 30^{\circ }\\ 180^{\circ }\\ 1 \end{array} \end{array}\right]$ at 14 GHz can be achieved. The perfect circular radiation peaks with radiation depressions are produced in both the propagating directions, and the maximum gain of vortex radiations are 15.4 dBi and 15.7 dBi. The VSWR of the meta-surface lens can keep less than 2 from 13.5 GHz to 14.5 GHz. On the other hand, *y*-polarized radiation beams of ${\left[\begin{array}{c} \theta_{i}\\ \varphi_{i}\\ l_{i} \end{array}\right]}_{i=1,2}=\left[\begin{array}{cc} \begin{array}{c} 30^{\circ }\\ 90^{\circ }\\ 1 \end{array} & \begin{array}{c} 30^{\circ }\\ 270^{\circ }\\ 1 \end{array} \end{array}\right]$ are well synthesized at 18 GHz. We can observe the maximum gain of vortex radiations are 17 dBi and 17.6 dBi. The VSWR of the meta-surface lens can keep less than 2 from 17.5 GHz to 18.5 GHz. When the meta-surface lens aperture becomes larger, the radiation will be more directive, and double the radiating aperture of the lens will lead to a 3 dB gain increase in general. The magnitude patterns and phase patterns of the *x*- and *y*-polarized vortex waves are demonstrated at 2000 mm away from the meta-surface lens with a scanning range of 600 × 600 ${\mathrm{mm}}^{2}$. The amplitude distributions show a circular distribution with central defect singularity, while the phase patterns show a 360° phase change for both OAM vortex beams around the center of the radiation aperture at 14 GHz as shown in Figure 4c. Meanwhile, Figure 4d shows the amplitude and phase distribution of the dual vortex beams at 18 GHz, where we can observe that the amplitude distributions both show a circular distribution with central singularity and the phase distributions all agree with our proposed OAM beams having mode numbers of l=1. Figure 4e,f demonstrate the mode purities of OAM beams with the Fourier relationship between the OAM spectrum [29,30] and the phase distribution of electric fields (E-fields) having the form of

(3) $$P\;=\;\frac{1}{2\pi }\int_{0}^{2\pi }\psi \left(\varphi \right)d\varphi e^{-jl\varphi }$$

where *P* and ψ refer to the OAM spectrum and phase value of E-fields, φ refers to the elevation angle, and *l* refers to the topological charges. We can observe that the dual beams with l=1 at 14 GHz are having the mode purity of 93%, 92%, and the dual beams with l=1 at 18 GHz are having the mode purity of 91%, 92%.

**Figure 5:**
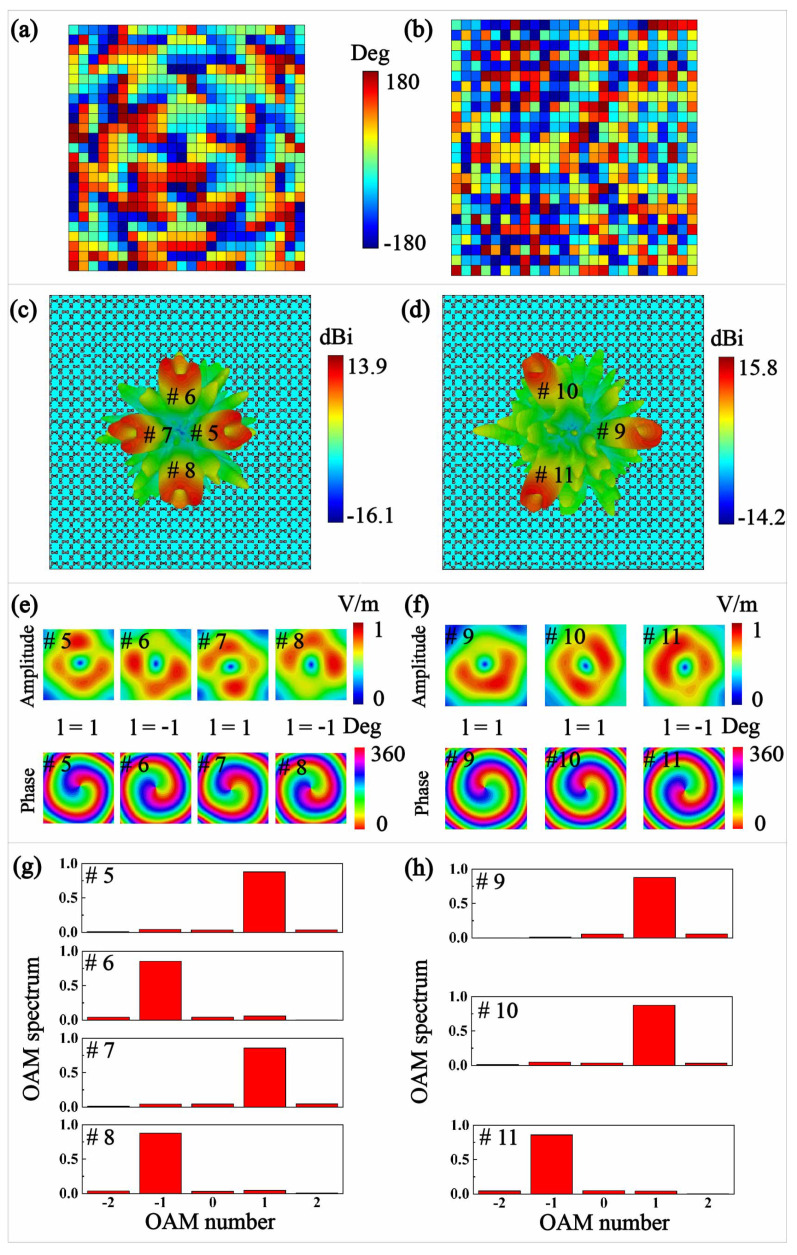
The multiple vortex beams with different polarization states from the laminated meta-surface lens at different frequencies. The phase distributions of the meta-lens at 14 GHz (**a**) and 18 GHz (**b**). The three dimension (3D) far-filed parents for *x*-LP quadruple vortex beams at 14 GHz (**c**) and *y*-LP triple vortex beams at 18 GHz (**d**). The amplitude patterns and phase patterns for *x*-LP quadruple vortex beams at 14 GHz (**e**) and *y*-LP triple vortex beams at 18 GHz (**f**). The mode purities of *x*-LP quadruple vortex beams at 14 GHz (**g**) and *y*-LP triple vortex beams at 18 GHz (**h**).

We continue to display the generation of triple vortex beams and quadruple vortex beams from the dual-band laminated meta-surface lens, as demonstrated in Figure 5, where the well synthesized four beams are directed in ${\left[\begin{array}{c} \theta_{i}\\ \varphi_{i}\\ l_{i} \end{array}\right]}_{i=1∼4}=\left[\begin{array}{cccc} 20^{\circ } & 25^{\circ } & 20^{\circ } & 25^{\circ }\\ 0^{\circ } & 90^{\circ } & 180^{\circ } & 270^{\circ }\\ 1 & -1 & 1 & -1 \end{array}\right]$ at 14 GHz and three beams in ${\left[\begin{array}{c} \theta_{i}\\ \varphi_{i}\\ l_{i} \end{array}\right]}_{i=1∼3}=\left[\begin{array}{ccc} 30^{\circ } & 30^{\circ } & 30^{\circ }\\ 0^{\circ } & 120^{\circ } & 240^{\circ }\\ 1 & 1 & -1 \end{array}\right]$ at 18 GHz. The corresponding phase distributions are calculated through superposing aperture fields by considering the beam directions and vortex modes at different frequencies. For the quadruple beam radiations at 14 GHz, the maximum gains of the *x*-polarized beams are 13.9 dBi, 13.8 dBi, 13.4 dBi, 13.9 dBi and the VSWR can keep less than 2 from 13.5 GHz to 14.5 GHz. The maximum gains at 18 GHz of the *y*-polarized radiations are 15 dBi, 15.1 dBi, 15.8 dBi and the VSWR can keep less than 2 from 17.5 GHz to 18.5 GHz. In the meantime, the magnitude patterns and phase patterns of the converged LP vortex beams are also demonstrated at 2000 mm away from the meta-surface lens with a scanning range of 600 × 600 ${\mathrm{mm}}^{2}$. All of the amplitudes show the circular distributions with a central defect singularity, while the phase patterns demonstrate 360° phase change for l=1 and −360° phase change for l=−1. The quadruple vortex beams with mode number l=1,−1,1,−1 at 14 GHz are having the mode purities of 88%, 86%, 86%, 88%, and the corresponding triple vortex beams with mode number l=1,1,−1 at 18 GHz are possessing the mode purities of 88%, 87%, and 86%, respectively.

**Figure 6:**
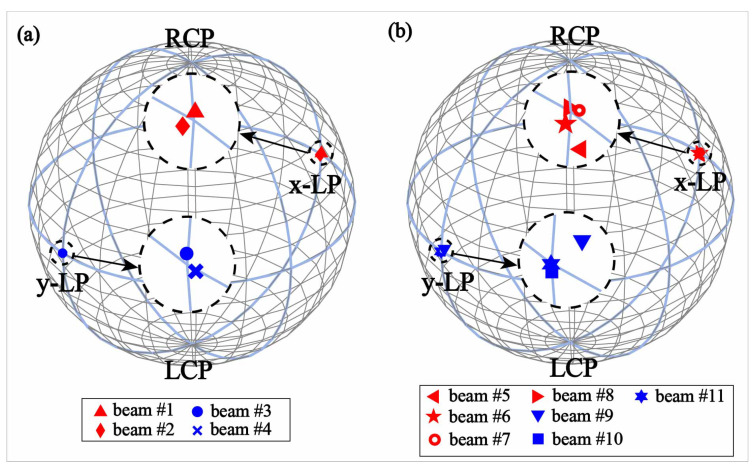
The demonstration of the corresponding polarization purity of the multiple OAM vertex beams on the Poincare sphere. The polarization purity of each beam in case I (**a**) and II (**b**), where the red and blue symbols represent the polarization purity of the beams at 14 GHz and 18 GHz, respectively.The LHCP is an abbreviation for left-handed circular polarization, and the RHCP is an abbreviation for right-handed circular polarization.

We employ ηP=S1S1S0S0 to evaluate the polarization purity of the radiating beams, where $S_{0}\;=\;E_{ox}^{2}\;+\;E_{oy}^{2}$ is the Stokes parameter describing the total intensity of the radiation beam, and $S_{1}\;=\;E_{ox}^{2}-E_{oy}^{2}$ is the Stokes parameter describing the preponderance of *x*-polarized waves over *y*-polarized waves. Clearly, the purity of *x*-polarized beams will be ideal when $\eta_{P}$ is close to 1, whereas the radiations will be pure of *y*-polarized beams when $\eta_{P}$ reaches −1. We can clearly observe from Figure 6 that the values of $S_{1}$ for all beams are distributed near east and west endpoints of the equator of the Poincare sphere, indicating that all the OAM vortex beams from the proposed laminated meta-surface lenses possess the merits of high polarization purity.

## 3. Conclusions

In conclusion, we have demonstrated the multiple OAM vortex beam synthesis with different radiating states at different frequencies through a laminated meta-surface lens. Through integrating the shared dual-polarized radiating aperture, high polarization purity multiple vortex beams of orthogonal polarized states can readily be generated at dual frequencies under the same excitation with compact and simplified lens construction. On the other hand, such a shared aperture design will also suffer the degradations of the radiating efficiency, and the radiation can be further improved by systematically optimizing the layout of the shared-aperture meta-surface arrays. As for the practical implementation, the manufacture of laminated circuit boards is very mature and the meta-surface lens we designed can be made by etching copper on Rogers RO4350B (ε=3.5, tanδ=0.003) and the multi-layer PCB boards. We expect our design of using laminated meta-surface lenses to generate different polarized multiple OAM vortex beams at different frequencies would offer a convenient way to build up a more advanced vortex beam communication system for more diversified capability.

## Figures and Tables

**Table 1:** The physical parameters of meta-surface lens.

Structural Parameters	Value	Structural Parameters	Value
$\alpha_{1}$	2.6 mm	*e*	8 mm
$\alpha_{1}$	1.3 mm	*f*	31.2 mm
$w_{1}$	1 mm	*g*	24.4 mm
$r_{1}$	0.4 mm	*k*	7.5 mm
$r_{2}$	0.8 mm	*p*	15.9 mm
$h_{1}$	1 mm	$\varepsilon_{1}$	3.5

## Data Availability

The data presented in this study are available from the corresponding author upon reasonable request.
